# Engineering and Exploiting Self-Driven Domain Wall Motion in Ferrimagnets for Neuromorphic Computing Applications

**DOI:** 10.1021/acs.nanolett.6c00181

**Published:** 2026-04-14

**Authors:** Jeffrey A. Brock, Aleksandr Kurenkov, David R. Lindenmann, Aleš Hrabec, Laura J. Heyderman

**Affiliations:** † Laboratory for Mesoscopic Systems, Department of Materials, 27219 ETH Zurich, 8093 Zurich, Switzerland; ‡ PSI Center for Neutron and Muon Sciences, 5232 Villigen PSI, Switzerland

**Keywords:** magnetic domain walls, neuromorphic computing, spintronics, ferrimagnets

## Abstract

Magnetic domain wall motion has recently garnered significant interest as a physical mechanism to enable energy-efficient, next-generation, brain-inspired computing architectures. However, realizing all behaviors required for neuromorphic computing within standard material systems remains a significant challenge, as these functionalities often rely on competing interactions. Here, we demonstrate how spontaneous domain wall motion in response to locally engineered lateral exchange coupling in transition metal–rare earth ferrimagnets can be leveraged to achieve numerous neuromorphic computing functionalities in devices with minimal complexity. Through experiments and micromagnetic simulations, we show how tuning the feature size, material composition, and chiral interaction strength controls the speed of self-driven domain wall motion. When integrated with current-induced spin–orbit torques, this control gives rise to behaviors essential for neuromorphic computing, including leaky integration and passive resetting of artificial neuron potential. These results establish locally engineered ferrimagnets as a tunable, scalable, and straightforward platform for domain wall-based computing architectures.

## Graphical abstract

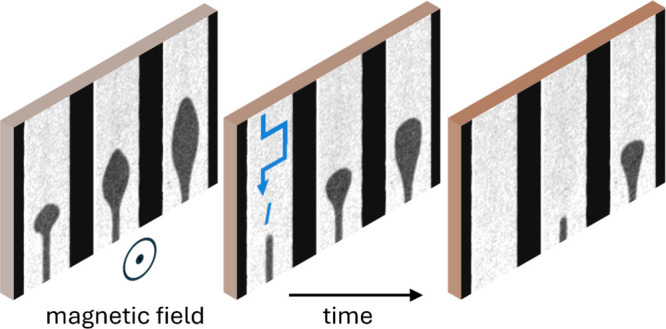

In an effort to realize more energy-efficient approaches to computing, the past several years have seen significant research invested in creating analogues of biological neural networks in condensed matter systems. Regardless of the physical property used to mimic neuron potentiation, an artificial neuron must simultaneously exhibit several capabilities, including (i) integration of input stimuli, (ii) leaking of potential between stimuli, and (iii) resetting back to an initial state after firing upon reaching a threshold potential.^1–5^ Spintronic systems are particularly interesting in this context, with tunable functionalities that can provide rich nonlinear dynamics and stochasticity while maintaining a high endurance.^6–9^ Among spintronic platforms for neuromorphic computing, magnetic domain walls (DWs) are exceptionally promising due to their ability to be moved and detected using electric currents. Additionally, the nonvolatility of DWs facilitates in-memory computing, avoiding the energy and time inefficiency associated with separation of memory and processing in traditional von Neumann architectures.^10^

To relate magnetic DW motion to neuromorphic computing, analogies can be drawn between the behaviors of DWs and those of biological neurons. Namely, the position of a DW is analogous to the potential energy of a neuron, the accumulated DW displacement in response to a series of external stimuli emulates the neuronal integration, and neuron firing corresponds to the DW reaching a specific position.^10–13^ To mimic biological neurons, the integration should be leaky, meaning that, without an input signal, the DW retreats with time.^14–17^ Additionally, a passive reset of the DW to its original state after firing is necessary.^10,18,19^ Given the diverse functionalities required of DW-based artificial neurons, designing materials and devices hosting competing interactions to enable all these behaviors in one device remains challenging.^11,20–22^

In parallel to the growing interest in using magnetic DWs for neuromorphic computing, transition metal–rare earth (TM-RE) ferrimagnetic alloys have regained interest because of their exceptional magnetization dynamics, which is useful for applications making use of extraordinarily fast and efficient domain wall motion.^23–25^ Ferrimagnets also generate low stray fields because of their low net magnetization, enabling denser device integration compared to ferromagnets.^26^ Furthermore, the ease with which the magnetically dominant sublattice of TM-RE ferrimagnets can be locally modified—using focused ion or electron beams, composition gradients, oxygen plasma, or laser annealing—has enabled new magnetic behaviors,^27–35^ including spontaneous DW motion in the absence of external stimuli.^36^

Here, we show that spontaneous DW motion arising from local control of the dominant magnetic sublattice in ferrimagnets provides the leak and reset behaviors necessary to construct artificial neurons. In addition, we demonstrate several patterning strategies to modulate these behaviors. We then integrate this control of spontaneous DW motion with current-induced spin–orbit torques (SOTs) to build neuronal structures that exhibit leaky signal integration and passive reset once the input signal—the applied current—is turned off. Our results show how the fast and efficient DW motion in TM-RE ferrimagnets, together with precise local patterning to control the key parameters, provides a straightforward-to-implement platform for neuromorphic computing.

To engineer spontaneous DW motion in our TM-RE ferrimagnet devices, we used direct-write laser annealing (DWLA)^34^ to create TM-dominant tracks within an RE-dominant Co_70_Gd_30_ film shown schematically in Figure 1a (see Supporting Information Section S1 for details). The exposed tracks have different widths, *w*_track_, defined in Figure 1c, ranging from 500 nm to 7 μm. To illustrate how this local patterning of ferrimagnetic properties yields self-driven DW motion in the track, we first apply an out-of-plane magnetic field, *H*, as shown in Figure 1a. If *H* exceeds the coercive field but remains weaker than the antiferromagnetic Co–Gd coupling (*J*_Co–Gd_), the net magnetizations (*M*_net_) of all regions align with those of *H*. To highlight this, the detailed magnetic configuration across the green plane in Figure 1a, consisting of two RE-dominant regions separated by a TM-dominant region, is shown in Figure 1b. This parallel configuration of *M*_net_ means that the magnetization of the Co (Gd) sublattice alternates when going from left to right, pointing down–up–down (up–down–up). As a result of this alternating configuration, DWs are stabilized at the interface between regions with different dominant sublattices (the light blue boundary in Figure 1a). Unlike in conventional magnetic materials, these domain states are stabilized by the applied magnetic field and are defined by a spatial variation in the sublattice magnetization orientation (as opposed to local *M*_net_).

**Figure 1:**
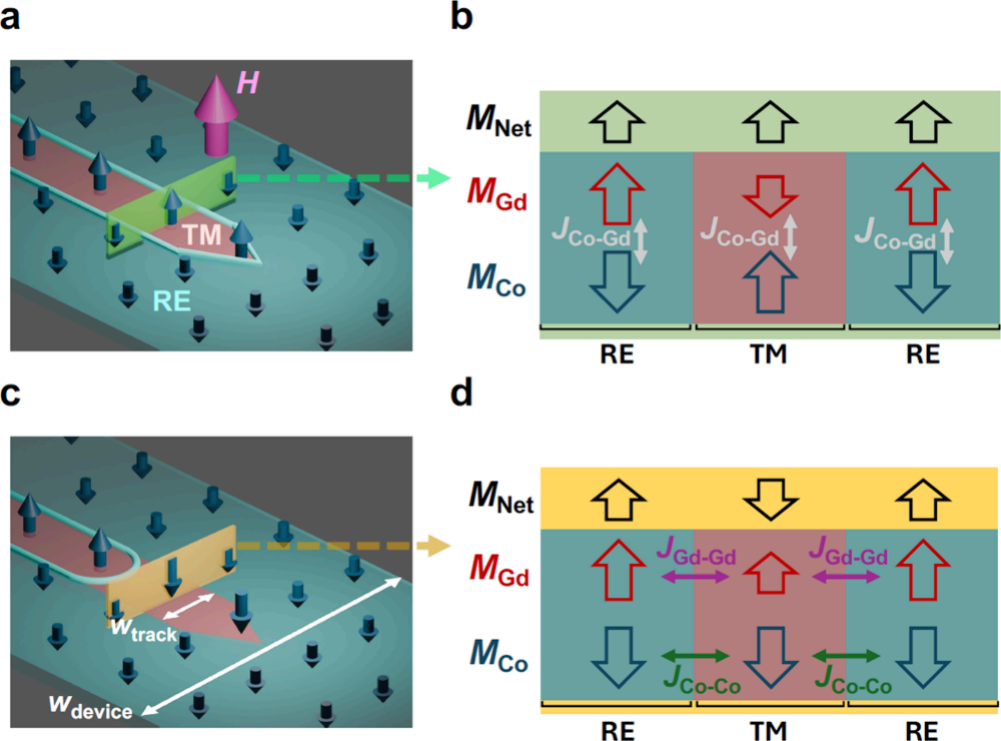
Engineered self-driven DW motion in TM-RE CoGd ferrimagnet tracks. (a) Schematic illustration of a TM-dominant track (pink inner area) within an as-grown RE-dominant device (teal outer area) created with DWLA. In an applied magnetic field *H* (magenta arrow), the net magnetizations of the TM- and RE-dominant regions *M*_net_ align parallel to *H*, while still satisfying the antiferromagnetic Co–Gd exchange interaction *J*_Co–Gd_ as highlighted in the cross-sectional view of the magnetic configuration across the green plane in (b). The DW between the TM- and RE-dominant regions is indicated by the light blue boundary in (a). (c) Schematic illustration of the domain configuration after *H* is removed, indicating the track and device width (*w*_track_ and *w*_device_, respectively). (d) Cross-sectional view of the magnetic configuration across the yellow plane in (c), demonstrating that, in the absence of *H*, there is a reversal in the net magnetization of the TM-dominant region to minimize the ferromagnetic Co–Co and Gd–Gd exchange energies (*J*_Co–Co_ and *J*_Gd–Gd_). The resulting antiparallel configuration of *M*_net_ corresponds to parallel alignment of the magnetization in the TM- and RE-dominant regions and a retreat of the light blue DW in (c). The dark blue arrows in (a) and (c) represent the Co magnetization, which is imaged with MOKE microscopy.

When *H* is removed (Figure 1c), the impact of the ferromagnetic Co–Co and Gd–Gd exchange interactions (*J*_Co–Co_ and *J*_Gd–Gd_, respectively) emerges. Specifically, *J*_Co–Co_ and *J*_Gd–Gd_ favor parallel alignment of the Co and Gd sublattice magnetizations so that, when *H* is removed, the lateral exchange coupling (LEC) across the boundaries between the TM- and RE-dominant regions promotes a spontaneous reversal of *M*_net_ in the TM-dominant region to minimize the ferromagnetic exchange energy as shown in Figure 1d, corresponding to a cross-sectional view across the yellow plane in Figure 1c. As we have shown previously, the LEC-induced magnetization reversal of a TM-dominant region with an apex-shaped end proceeds in a specific sequence:^36^ First, *M*_net_ is reversed within the apex of the TM-dominant region (Figure 1c) where, as an interfacial interaction, the LEC strength is maximized. This reversal proceeds through motion of the DW that previously existed at the edges of the apex region (light blue boundary in Figure 1a) into the TM-dominant region (Figure 1c), observed as DW motion along the track. The curvature of the DW within the TM-dominant region (Figure 1c) is a direct result of the fact that the LEC originates at the interface between the TM- and RE-dominant regions.^36^ This DW section then propagates down the TM-dominant track, yielding parallel alignment of *M*_net_ across like sublattices (see Figure 1d).

The effect of LEC can be quantitatively described in terms of an effective exchange coupling magnetic field, *H*_LEC_, which drives the DW motion. This magnetic field can be expressed as^36,37^

1 $$\mu_{0}H_{\mathrm{LEC}}=\frac{\lambda_{\mathrm{DW}}}{M_{\mathrm{tot}}w_{\mathrm{track}}}$$

where *M*_tot_, *w*_track_, and $\lambda_{\mathrm{DW}}\approx \sqrt{AK_{\mathrm{eff}}}$ are the net magnetization, width of the track, and domain wall energy density given by exchange constant *A* and effective anisotropy *K*_eff_. As the equation suggests, smaller track widths result in larger effective exchange fields and thus faster DW velocities.

While we previously cataloged the impact of LEC through magnetometry measurements,^36^ we now determine the material factors that govern LEC-driven DW motion and how this DW motion can be coupled with SOTs to yield behaviors relevant for neuromorphic computing. We show that the speed of LEC-driven DW motion directly determines the leak and reset behaviors, as well as the integrate and fire functionalities, essential to neuromorphic computing with magnetic DWs. Therefore, a precise understanding of how the speed of spontaneous DW motion can be tuned is critical for developing DW-based artificial neurons. Accordingly, we denote the velocity of self-driven DWs as *v*_leak/reset_ throughout this work.

To observe how *w*_track_ impacts *v*_leak/reset_, we began by initializing a parallel alignment of *M*_net_ across the TM- and RE-dominant regions using a +200 mT out-of-plane magnetic field as shown schematically in Figure 1a. A magneto-optic Kerr effect (MOKE) image of the magnetic configuration stabilized in this magnetic field for five tracks with different *w*_track_, ranging from 2 to 4 μm, is shown in Figure 2a. Because the white-light illumination we use for MOKE imaging is most sensitive to the Co sublattice,^23,38,39^ the contrast of the track (surrounding region) with the Co magnetization pointing out of (into) the plane appears dark (bright). After the magnetic field was removed, we recorded MOKE images at 25 Hz (Supporting Video 1). Within 0.44 s of removing the magnetic field, almost half of the track with *w*_track_ = 2 μm has reversed, whereas smaller portions of the tracks with larger *w*_track_ have reversed (Figure 2b). 0.72 and 1.16 s after the magnetic field was removed (Figures 2c and 2d, respectively), the DW displacements remain inversely related to *w*_track_. To quantify how *w*_track_ impacts the speed of LEC-propelled DWs, we determined *v*_leak/reset_ for the different *w*_track_ values given by the solid blue squares in Figure 2e (see Supporting Information Section S2 for details on the velocity calculations). We find that *v*_leak/reset_ decays with increasing *w*_track_, such that the LEC becomes too weak to promote spontaneous DW motion when *w*_track_ ≥ 7 μm. Furthermore, we find that this inverse relationship between *w*_track_ and *v*_leak/reset_ is the same for a reversed polarity of the initialization magnetic field (open blue squares in Figure 2e). The observed dependence of *v*_leak/reset_ on *w*_track_ aligns with prior observations that narrower tracks, being more interfacial in character, exhibit stronger LEC,^36^ as described by Equation 1. Here, we show for the first time that a stronger LEC leads to faster self-driven DW motion.

**Figure 2:**
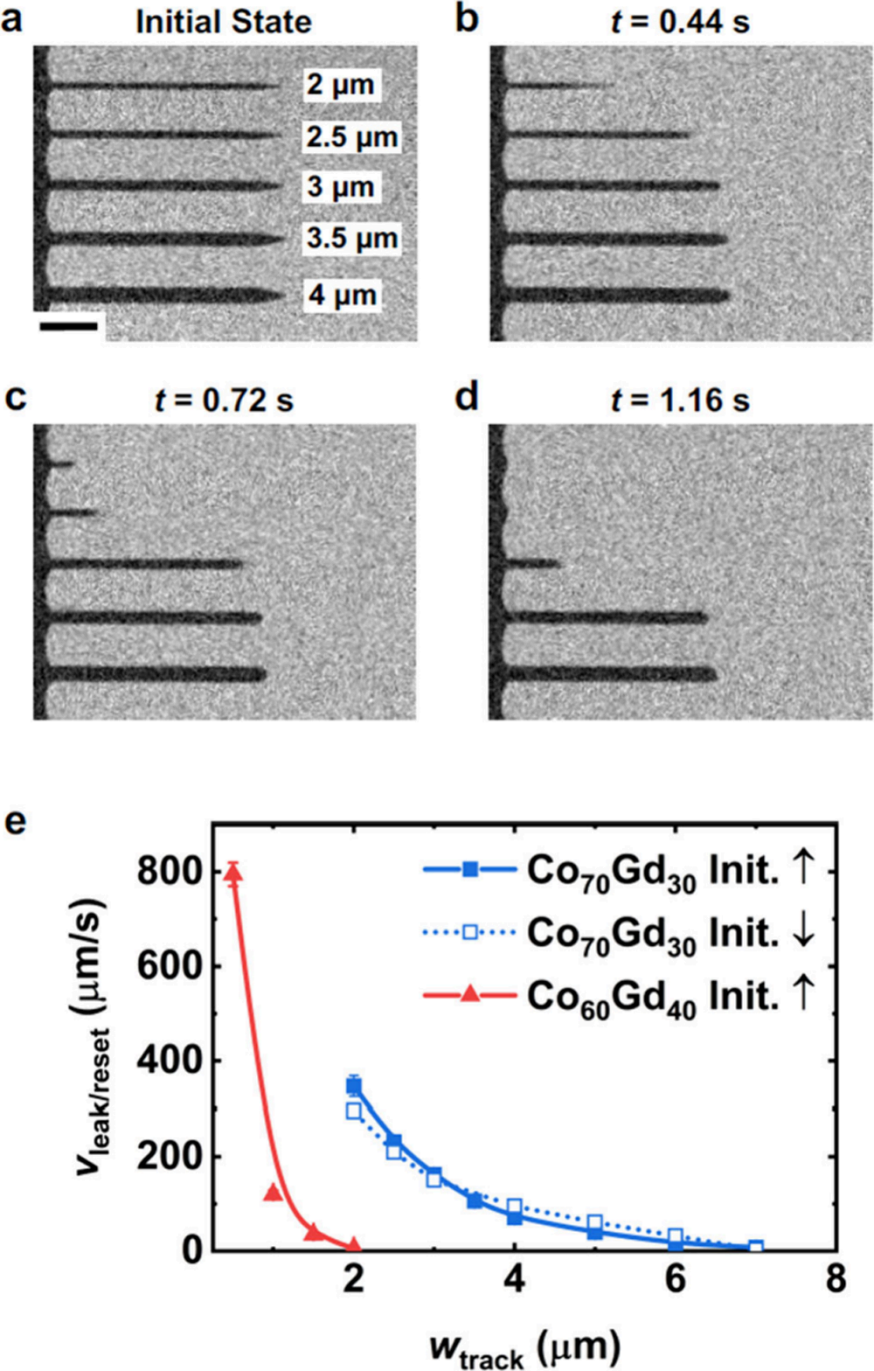
Controlling self-driven DW leak and reset velocities by modifying the track width and ferrimagnet composition. (a–d) Polar MOKE images of the domain state in the Co_70_Gd_30_ sample while a +200 mT out-of-plane magnetic field was applied to initialize the sample (a), then at 0.44 s (b), 0.72 s (c), and 1.16 s (d) after the magnetic field was removed (scale bar = 15 μm). *w*_track_ for each track is indicated in (a). In (a–d), dark (bright) contrast is associated with regions where the Co sublattice is magnetized up (down), corresponding to the TM-dominant (RE-dominant) regions. (e) The average DW leak and reset velocity *v*_leak/reset_ as a function of *w*_track_ is shown for the Co_70_Gd_30_ and Co_60_Gd_40_ samples (blue squares and red triangles, respectively). The lines are guides for the eye. For the Co_70_Gd_30_ sample, data for initialization in a −200 mT magnetic field is also provided (open blue squares). See Supporting Information Section S2 for details on the determination of *v*_leak/reset_ and the error bars.

While we see that *w*_track_ strongly influences the speed of self-driven DWs, it is also essential to determine how intrinsic material properties, such as the TM-RE ferrimagnet composition, affect the LEC-driven DW motion. To accomplish this, we fabricated a Co_60_Gd_40_ sample, so with a 10% increase in Gd concentration (see Supporting Information Section S1 for details). The Co_60_Gd_40_ sample was RE-dominant with perpendicular magnetic anisotropy in the as-grown state and received the same DWLA treatment as that of the Co_70_Gd_30_ sample. Using the same experimental procedure as above, we find that, while the value of *w*_track_ above which LEC cannot induce DW motion in the Co_70_Gd_30_ sample is 7 μm, this value drops to 2 μm in the Co_60_Gd_40_ sample (compare solid blue squares and red triangles in Figure 2e). In previous work, it was shown that a Co_60_Gd_40_ sample can have a net exchange stiffness ∼60% lower than a Co_70_Gd_30_ sample,^40–42^ which can result in weaker LEC and therefore lower DW velocities. Indeed, it can be seen from Equation 1 that the reduced exchange coupling results in lower DW energies, which in turn result in lower effective magnetic field driving the DW. Also, increasing the Gd content reduces the saturation magnetization of the as-grown film at room temperature by ∼20% (see Figure S1 in the Supporting Information), reducing dipolar interactions between the TM- and RE-dominant regions, potentially slowing LEC-induced DW motion—a possibility that we later demonstrate. While precisely distinguishing the relative impact of these two effects on the observed DW dynamics is nontrivial, Figure 2e nonetheless illustrates that changing the ferrimagnet composition provides another means of controlling LEC-driven DW motion. In addition, we have investigated how the limits in spatial resolution of the laser annealing technique and variations of ambient temperature affect the device performance (Supporting Information Section S3). Our understanding of how material properties affect LEC is crucial to the scalability of self-driven DW motion, as it allows the leak and reset dynamics of artificial neurons to be tuned for specific device sizes.

Since any potential technological application of magnetic DW motion inevitably requires maximizing areal density through device miniaturization, we now examine how LEC-driven DW motion is impacted by reducing the size of the RE-dominant region surrounding the TM-dominant tracks. To accomplish this, we prepared devices that feature TM-dominant tracks of fixed *w*_track_ = 5 μm while varying the width of the surrounding device *w*_device_, defined in Figure 1c, from 45 down to 9 μm (Figure 3a and Supporting Video 2). Repeating the same initialization procedure as before, we determined *v*_leak/reset_ as a function of *w*_device_ (blue circles in Figure 3b), finding that, for a fixed *w*_track_, there is a strong inverse relationship between *w*_device_ and *v*_leak/reset_. To understand the observed relationship between *w*_device_ and measured *v*_leak/reset_, we micromagnetically simulated the LEC-induced DW dynamics. Full details on the simulation parameters are provided in the Supporting Information Section S4, including the interfacial Dzyaloshinskii–Moriya interaction (iDMI) present at the Pt/CoGd interface.^24,43,44^ The simulated regions correspond to DW segments similar to the one enclosed by the blue box in Figure 3a. From these simulations, we extracted *v*_leak/reset_ as a function of *w*_device_ (blue circles in Figure 3c), finding a trend similar to the experimental results of Figure 3b. To determine the origins of this inverse relationship between *w*_device_ and *v*_leak/reset_, we consider the magnetic configuration of our devices 3 ns after the simulation began. Inspecting the cross-sectional view of the simulation with *w*_device_ = 400 nm shown in Figure 3d (corresponding to the region indicated with the dashed red line in Figure 3a), the magnetization rotates counterclockwise as one moves from the left to right across the DWs; this rotation is commensurate with the left-handed Néel-type chirality promoted by the iDMI at the Pt/CoGd interface.^24,43,44^ Additionally, at the edges of the device, the magnetization cants away from the *z*-axis, resulting in an additional magnetization component along the +*x*-axis (−*x*-axis) at the left (right) edge. The opposite canting at the left and right edges matches the DW chirality stabilized by the iDMI.^45–48^

**Figure 3:**
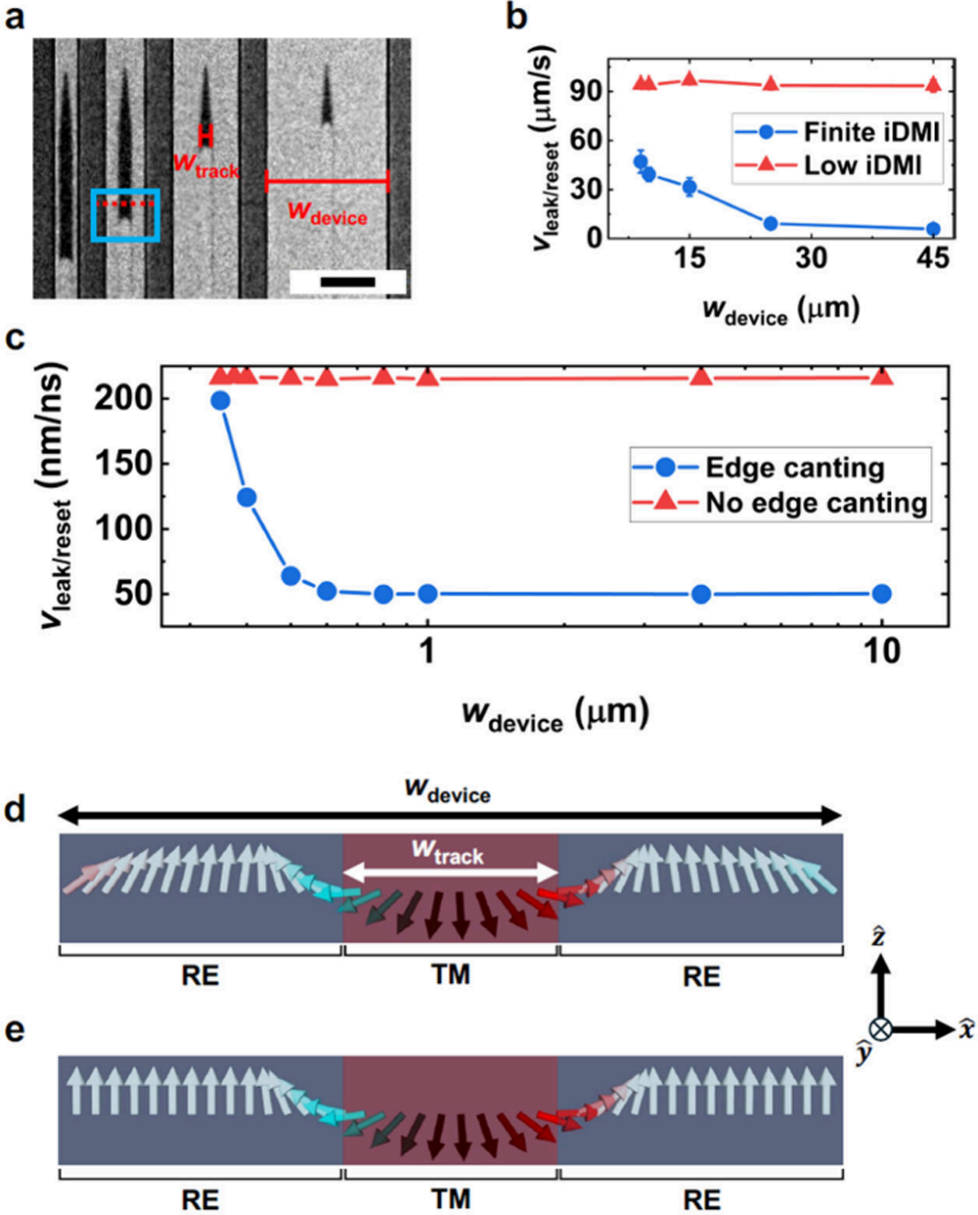
Joint influence of device size and iDMI on DW leak and reset behaviors. (a) Polar MOKE image of the domain state present in a Co_70_Gd_30_ sample with finite iDMI strength that has been lithographically patterned into devices of varying width *w*_device_, with *w*_track_ = 5 μm, 2.52 s after the initializing magnetic field was removed. For this measurement, the background image subtracted from the displayed image was collected while applying a −200 mT magnetic field (scale bar = 20 μm). (b) The DW leak/reset velocity *v*_leak/reset_ as a function of *w*_device_ for the Co_70_Gd_30_ samples with finite and low iDMI. Details on how *v*_leak/reset_ was determined are provided in Section S2 of the Supporting Information. (c) *v*_leak/reset_ as a function of *w*_device_ obtained from micromagnetic simulations for systems with and without chiral edge canting. (d, e) Cross-sectional views of the magnetic configuration along paths similar to the red dashed line in (a) for simulations with (d) and without (e) chiral edge canting. The simulations from which (d) and (e) were taken are provided in Figure S7 of the Supporting Information.

Altogether, our simulations reveal that the chirality of both the DW and the edge canting promoted by the iDMI dictates a slight yet discernible head-to-head arrangement of the magnetization between the edges of the device and the magnetization in the DWs. Such head-to-head configurations are known to incur a magnetostatic energy penalty,^49^ and one way to reduce the energy is for the end section of the DW to propagate through the track, thereby eliminating the DW from the system. As *w*_device_ is reduced and, hence, the distance between the side sections of the DW and device edges is also lowered, the DW energy density increases, thus providing an additional impetus for DW motion beyond that provided by LEC alone (see Figure S7 in the Supporting Information).

To confirm the roles that DW chirality and edge canting play in modulating the dynamics of LEC-driven DWs in patterned devices, we repeated the simulations with the magnetization of the RE-dominant regions fixed out-of-plane, where the suppressed chiral edge canting can be seen in Figure 3e. Because freezing the magnetization of the RE-dominant region in an out-of-plane configuration prevents the DW and edge magnetization profiles from assuming a head-to-head configuration (see the cross sectional view of a system with edge canting disabled in Figure 3e), the DW energy density becomes independent of *w*_device_ (see Figure S7 in the Supporting Information). As a result, there is no appreciable change in the simulated *v*_leak/reset_ with *w*_device_ when the magnetization in the RE-dominant region is frozen (red triangles in Figure 3c). To experimentally validate this link between the iDMI, *v*_leak/reset_, and *w*_device_, we fabricated a sample with weaker iDMI, and thus a weaker degree of edge canting, also finding no pronounced change in *v*_leak/reset_ with *w*_device_ as shown by the red triangles in Figure 3b (see Supporting Information Section S1 for sample details).

Having demonstrated how patterned track and device width, TM-RE composition, and iDMI affect the LEC-driven motion of DWs, we now apply this tunability to yield functionalities relevant to neuromorphic computing. Specifically, we now combine in a single device SOT-induced DW motion along the track with LEC-driven spontaneous DW motion in the opposite direction. To obtain SOT-induced DW motion, a charge current is passed through our devices. Here, the spin current generated via the spin-Hall effect in the Pt layer exerts SOTs on the CoGd layer, which, coupled with the left-handed chiral Néel-type DWs stabilized by the iDMI originating from the Pt/CoGd interface, results in DW displacement parallel to the current.^24,44,50^

To implement the leaky integration and reset functionalities in our materials, we used the device structure illustrated in Figure 4a. Leveraging our understanding of how *w*_track_ governs *v*_leak/reset_, we patterned a TM-dominant region with a spatially varying *w*_track_ within a lithographically defined device designed to foster the current densities necessary for SOT-induced DW motion (Figure 4a). Specifically, the track contained an ellipse-shaped trap region where *w*_track_ > 7 μm, thus inhibiting spontaneous DW motion in response to LEC. In addition, within the trap region, *w*_device_ was expanded to minimize the current density within the trap, reducing the SOTs below the threshold required to move the DW within the trap region. Our device then operates as follows: After initializing the magnetic configuration in a +200 mT magnetic field, removing the magnetic field leads to LEC-induced DW propagation in the TM-dominant region from right to left until *w*_track_ begins to expand, becoming too large to promote spontaneous DW motion, thus pinning the DW (Figure 4b). On applying an electrical current pulse from left to right, SOTs displace the DW to the right (Figure 4c). The electrical current pulse parameters are listed in Figure 4. This SOT-induced DW motion emulates neuronal signal integration.^10–13^ However, on moving rightward from the trap area, the DW moves to a region where *w*_track_ = 3.5 μm, a value small enough to promote the leftward LEC-induced motion. As such, during the interval between two current pulses, the spontaneous DW motion back toward the trap area mimics a neuron leaking potential (Figure 4d).^14–17^ In the context of neuromorphic computing using magnetic DWs, neuron firing occurs when a DW reaches a defined position in the device.^10–13^ In the present device, where the LEC and SOT-induced DW motion oppose each other, the firing behavior is determined by the balance between integration and leak dynamics.

**Figure 4:**
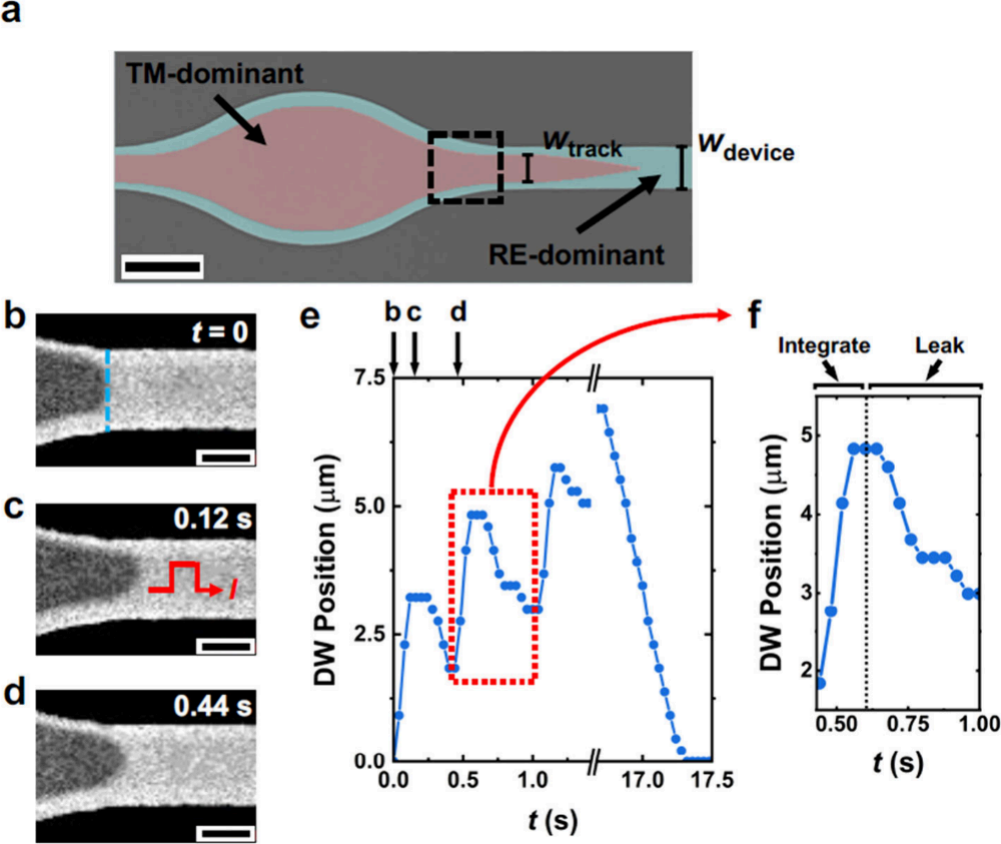
Leaky integration and passive reset of a DW neuron. (a) Optical microscopy image of the artificial neuron structure with a false color overlay indicating the TM- and RE-dominant regions in pink and teal, respectively (scale bar = 10 μm). (b–d) Polar MOKE images of the domain state present in a Co_70_Gd_30_ sample after the initializing magnetic field was removed (b), after the first current pulse was applied (c), and immediately before the second current pulse was applied (d). Scale bar: 5 μm. The black dashed box in (a) roughly indicates the area corresponding to the MOKE images in parts (b–d). The pulse sequence consisted of 120 μs-long square pulses of current density 1 × 10^10^ A/m^2^ applied at a repetition rate of 2 Hz, with the direction of the current flow *I* indicated in (c). (e) The DW position as a function of time while current pulses were applied to the device, with the origin defined by the vertical dashed blue line in (b). The arrows show the times corresponding to the images in (b, c, and d). (f) A more detailed view of the leaky integration behavior of our DW neuron, corresponding to the data enclosed by the red dashed box in (e).

To better illustrate the leaky integration and passive reset behavior of our system under the joint influence of SOTs and LEC, we plot the DW position as a function of time over many current pulses in Figure 4e (determined from the data shown in Supporting Video 3), where the initial pinned position is defined as the origin (blue dashed line in Figure 4b). Furthermore, once the current pulses are stopped (at *t* = 16.8 s), LEC returns the DW toward the origin, passively resetting the neuron potential.

Notably, our material platform also supports integrate-and-fire behavior when LEC- and SOT-induced DW motions act cooperatively. Here, we demonstrate how the length of a trap region *l*_trap_, defined in Figure 5a, in which *w*_track_ is wide enough to prevent spontaneous DW motion, can be used to adjust the integration behavior of DW-based neuronal structures. These devices again feature ellipse-shaped trap regions where *w*_track_ is greater than 7 μm, so as to prevent LEC-driven DW motion in response to LEC. The design principle of these devices is that, within the trap regions, displacement of a DW solely in response to the SOT generated by the current pulses emulates a neuron integrating signal. Varying *l*_trap_ from 20 to 40 to 70 μm (from left to right in Figure 5a) provides a means to adjust the DW displacement that must be integrated before the DW exits the trap regions. Outside the traps, *w*_track_ was fixed to 4.5 μm, which is a value small enough to ensure a spontaneous LEC-driven DW motion. This allows the DW, once displaced from the trap by SOT, to spontaneously propagate to the end of the track, where its arrival signifies neuronal firing.^11,12,51^

**Figure 5:**
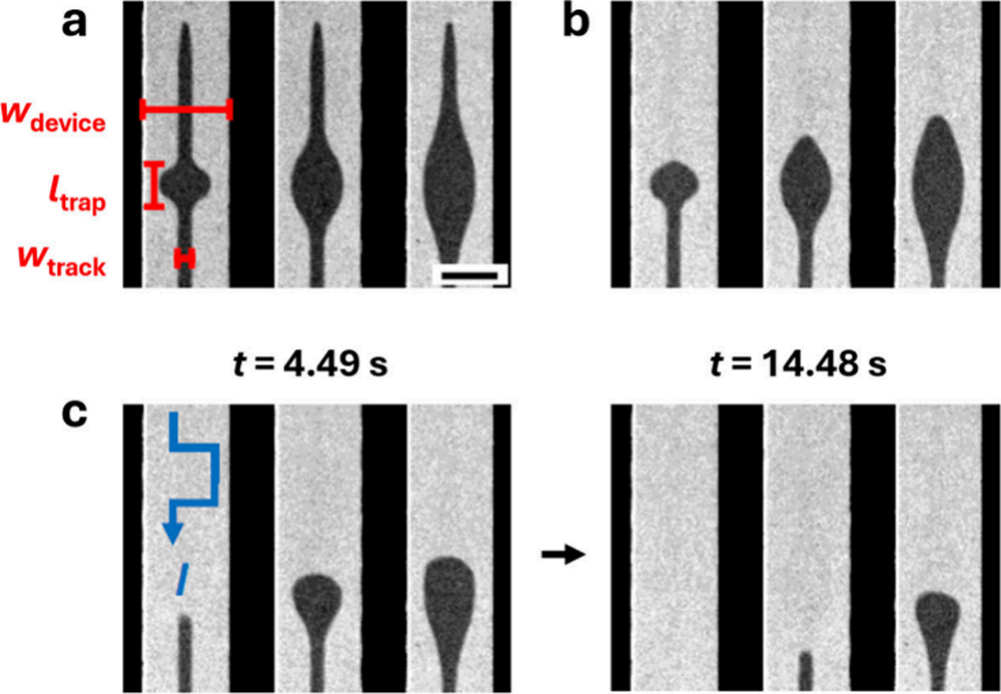
Integrate and fire functionality. (a) Polar MOKE images of the domain state present in a Co_70_Gd_30_ sample engineered to have TM-dominant tracks with elliptical trap regions with trap lengths *l*_trap_ of 20 μm, 40 μm, and 70 μm (left to right, respectively) while a +200 mT perpendicular magnetic field was applied to initialize the sample (scale bar = 20 μm). (b) The magnetic configuration some time after the initializing magnetic field was removed. (c) The domain configurations 4.49 s (left) and 14.48 s (right) after current pulses were applied to the sample. The pulse sequence consisted of 120 μs-long square pulses of current density 1 × 10^10^ A/m^2^ applied at a repetition rate of 2 Hz, with the direction of the current flow *I* indicated in blue.

We now demonstrate these integration and firing functions, first initializing the net magnetization of the TM- and RE-dominant regions in a parallel state by applying a +200 mT perpendicular magnetic field to the device (Figure 5a). Upon removal of the magnetic field, magnetization reversal of the upper part of the TM-dominant region of the track proceeds via DW motion, with the DW nucleated in the apex region and subsequently moving through the device until becoming pinned at the top ends of the traps (Figure 5b). Next, current pulses were applied simultaneously to all three track structures, enabling concurrent imaging of how the DWs move through the traps in response to the same current pulse amplitude.

After the current pulses were applied for 4.49 s, the gradual accumulation of DW displacement over the course of several current pulses mimics the neuronal integration process (Figure 5c). When the DW is fully displaced from the trap and into a region where *w*_track_ is low enough to promote spontaneous DW motion, the DW rapidly propagates to the lower end of the track, a response that corresponds to firing of the artificial neuron upon reaching a threshold potential. In the devices with *l*_trap_ > 20 μm, SOT alone is insufficient to displace the DWs within the same time interval of 4.49 s, and the DWs remain confined to the portion of the track where *w*_track_ is larger. As a result, the devices with longer trap regions require more DW displacement before they fire (Figure 5c). As such, for a given stimulus, the integration required for a DW to traverse the trap is intimately linked to our ability to locally control the action of the LEC through *w*_track_.

Having shown how the engineered presence of LEC enables leaky integration and self-resetting functionalities essential for neuromorphic computing using magnetic DWs, we acknowledge that several desirable behaviors have yet to be realized in our systems. For example, the efficiency and accuracy of neural networks are greatly improved in systems exhibiting lateral inhibition, meaning that only the signal from the most responsive neuron is propagated forward through the network, while the output of competing neurons is suppressed (the winner-takes-all principle).^11,51,52^ Furthermore, an ability to modify the interaction strength between neurons (the so-called synaptic weight) during operation is essential.^10,22,53^ These functionalities rely on the ability to locally and temporarily modulate the strength of the LEC in our materials. For this, recent progress in the rapidly growing field of magnetoionics has shown that ionic gating can reversibly tune the magnetic properties of TM-RE ferrimagnets, including the exchange coupling strength and magnetically dominant sublattice,^54^ thus enabling on-demand, reversible control of LEC. For example, solid-state hydrogen gating could be used to control the strength of the LEC coupling, and even to completely suppress it, on the time scales down to a few tens of μs. These advances in materials control make lateral inhibition and synaptic weighting in ferrimagnet-based neuromorphic architectures increasingly within reach.

In conclusion, we have demonstrated how, for a neuron device consisting of a transition-metal dominant ferrimagnetic track coupled to a rare-earth-dominant device region, the size and shape of magnetically patterned regions, composition of ferrimagnetic materials, and presence of iDMI enable regulation of the leak and reset rates of artificial neuron devices. By integrating the control of spontaneous domain wall motion with current-induced SOTs, we have realized critical behaviors relevant to neuromorphic computing applications, including leaky integration and a passive reset of the domain wall position back to the initial state. Our device structure is easily implemented and scalable, leveraging materials grown via high-throughput, room-temperature sputter deposition, patterned through a single-step laser-induced process, and compatible with next-generation stimuli such as SOTs. Our findings establish an important foundation for exploiting lateral exchange coupling effects for next-generation magnetic domain wall-based bioinspired computing architectures.

## Supplementary Material









## Notes

Details of sample fabrication and processing, iDMI measurements, room-temperature magnetometry data for the Co_70_Gd_30_ and Co_60_Gd_40_ samples, domain wall width estimations, description of the MOKE imaging procedure, details of the micromagnetic simulations, the simulated dependence of DW velocity on the strength of exchange stiffness, the simulated energy density of DWs in systems with and without chiral edge canting (PDF)
Video 1: Domain wall motion as a function of *w*_device_ (AVI)
Video 2: Domain wall motion as a function of *w*_track_ (AVI)
Video 3: Leaky integration and passive reset in the artificial neuron (AVI)
