# Supplementary material for: Engineering and Exploiting Self-Driven Domain Wall Motion in Ferrimagnets for Neuromorphic Computing Applications
Source: Nano Lett. 2026 Apr 14;26(16):5426–33. doi: 10.1021/acs.nanolett.6c00181 (PMC13133903; doi:10.1021/acs.nanolett.6c00181)
Supplement: Supplementary file 1 [file nl6c00181_si_001.pdf]

Supporting Information

Engineering and Exploiting Self-Driven Domain Wall Motion in Ferrimagnets for Neuromorphic Computing Applications

Jeffrey A. Brock[1,2*], Aleksandr Kurenkov[1,2], David R. Lindenmann[1,2], Aleš Hrabec[1,2*], Laura J. Heyderman[1,2]

[1]Laboratory for Mesoscopic Systems, Department of Materials, ETH Zurich, 8093 Zurich, Switzerland.
[2]PSI Center for Neutron and Muon Sciences, 5232 Villigen PSI, Switzerland.

S1 Sample preparation

Thin film heterostructures with the composition Ta (2 nm)/ Pt (5 nm)/ Co_xGd_{1-x} (6 nm)/ Ta (4 nm) were deposited onto thermally oxidized Si substrates (oxide thickness = 300 nm) by magnetron sputtering. All layers were sputtered in an Ar pressure of 0.4 Pa. The Ta layers were deposited using an RF power of 100 W, while Pt, Co, and Gd were deposited using DC powers of 100 W, 50 W, and 24 W, respectively. Deposition rates for each material were calibrated by growing reference samples for 10 minutes and measuring their thickness using x-ray reflectivity. The Co_xGd_{1-x} alloy layers were deposited by co-sputtering Co and Gd, with the stated atomic fractions determined by the sputtering powers used. In-plane superconducting quantum interference device vibrating sample magnetometry (SQUID-VSM) measurements indicate that the $Co_{70}Gd_{30}$ and $Co_{60}Gd_{40}$ films, each 6 nm thick, have saturation magnetizations of 25 kA/m and 20 kA/m, respectively (Fig. S1). Both samples have an anisotropy field of approximately 0.75 T.

To determine the effect of lowering the iDMI energy density on LEC-induced DW motion, a $Co_{70}Gd_{30}$ sample of the composition Ti (4 nm)/ $Co_{70}Gd_{30}$ (8.5 nm)/ Ti (4 nm) was prepared, with the Ti deposited using a 100 W sputtering power. The $Co_{70}Gd_{30}$ layer in this sample was made slightly thicker to achieve perpendicular magnetic anisotropy (PMA) through bulk anisotropy, as interfacial contributions to PMA are suppressed in this heavy-metal-free composition.

The dominant magnetic sublattice in our CoGd samples (which were RE-dominant in the as-grown state) was locally modified by direct-write laser annealing using a laser fluence of 1.4 J/cm^2. Full details on the direct-write laser annealing technique and its impact on the properties of TM-RE ferrimagnets can be found in Ref. [1]. The size of the RE-dominant region surrounding the TM-dominant tracks was controlled using UV photolithography and ion beam etching. For this, a Heidelberg Instruments DWL 66+ system was used to pattern a mask in a layer of S1813 photoresist that was spin-coated onto the sample. After removing the exposed resist regions in a TMAH-based developer, an Ar^+ ion beam was used to etch the regions not covered by photoresist down to the substrate.

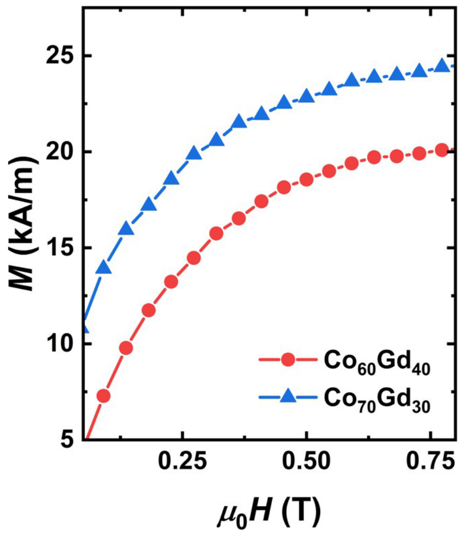

Figure S1: In-plane SQUID-VSM measurements of the $Co_{70}Gd_{30}$ and $Co_{60}Gd_{40}$ samples (blue triangles and red squares, respectively). The error bars for the magnetization *M* are smaller than the symbol size.

S2 MOKE microscopy of domain wall dynamics

A magneto optic Kerr effect (MOKE) microscopy system manufactured by Evico Magnetics GmbH was used to collect images capturing DW motion in our devices. The microscope employs white LED illumination, which provides sensitivity to the orientation of the Co sublattice magnetization in our CoGd films [2–4]. A reference image was taken in a -200 mT out-of-plane magnetic field before applying a +200 mT initialization magnetic field. This reference image was subsequently subtracted from the recorded images of the domain wall dynamics in order to improve image contrast.

Any deviations from this protocol are noted in the main text. For each w_{track} or w_{device} considered, the initialization and measurement process was repeated five times, with the velocities averaged over the five trials. Error bars correspond to the standard error in average velocity over the five trials. Electrical current pulses were applied to the samples using an AVTECH AVR-E3-B-W1 pulse generator. The magnitude of the electrical current pulses supplied to the devices was monitored using an oscilloscope connected along the ground path.

S3 Domain wall velocities in annealed tracks

Effect of micromagnetic parameters

The domain wall dynamics is directly connected with the domain wall width Δ, which can be determined from the following expression

$$\Delta = \sqrt{\frac{A}{K_{\text{eff}}}}, \qquad (1)$$

where K_{eff} is the effective anisotropy and A the composition-dependent exchange interaction. The anisotropy field $\mu_0 H_{\text{k}}$, required to determine effective anisotropy, was determined using SQUID-VSM (see Fig. S1) to be:

$$Co_{70}Gd_{30}\text{: } K_{\text{eff}} = \frac{1}{2}\mu_0 H_k M_s = 9.4 \text{ kJ/m}^3,$$
$$Co_{60}Gd_{40}\text{: } K_{\text{eff}} = \frac{1}{2}\mu_0 H_k M_s = 7.4 \text{ kJ/m}^3.$$

Given the uncertainty in the exchange energies for (i) $Co_{70}Gd_{30}$: $A = 5 - 9$ pJ/m and (ii) $Co_{60}Gd_{40}$: $A = 2 - 4$ pJ/m, we estimate the DW width to be $\Delta_{70/30} = 27\ \mathrm{nm}\ \pm 4\ \mathrm{nm}$ and $\Delta_{60/40} = 19\ \mathrm{nm}\ \pm 4\ \mathrm{nm}$, respectively.

To quantitatively evaluate samples with strong and weak iDMI, we performed measurements based on asymmetric domain expansion on samples with composition Pt/CoGd/Ta (strong iDMI) and Ti/CoGd/Ti (weak iDMI). Here, we drive the domain expansion with a fixed out-of-plane magnetic field while sweeping the in-plane magnetic field. Assuming that the domain wall energy is isotropic [5-7], the case where the applied in-plane magnetic field opposes and compensates for the internal effective DMI corresponds to the minimum domain wall velocity. It can be seen from Fig. S2(a) and (b) that this minimum domain velocity can be determined from fitting the data with the creep law in the presence of an in-plane magnetic field [7] shown by the red curves. This yields $\mu_0 H_{\mathrm{DMI}} = +220\ \mathrm{mT} \pm 10\ \mathrm{mT}$ for the Pt/ Co_{70} Gd_{30}/Ta sample and $\mu_0 H_{\mathrm{DMI}} = -42\ \mathrm{mT} \pm 5\ \mathrm{mT}$ for the Ti/ Co_{70} Gd_{30}/Ti sample.

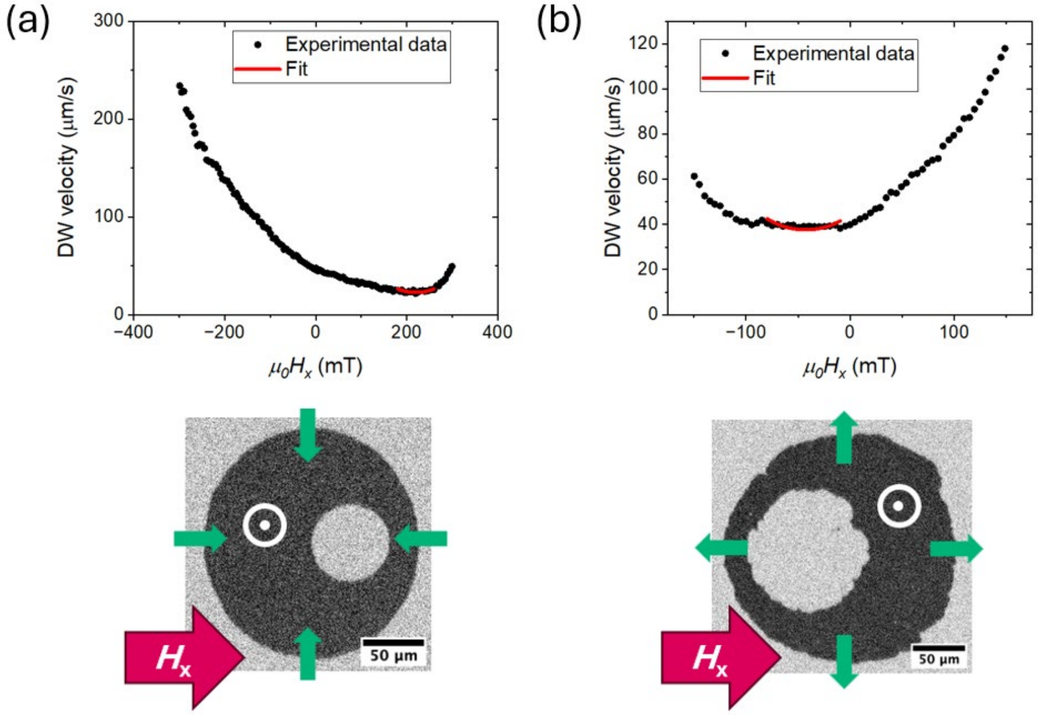

Figure S2: Upper panel: Domain wall velocities as a function of the applied in-plane magnetic field for (a) Pt/Co_{70} Gd_{30}/Ta and (b) Ti/$Co_{70}Gd_{30}$/Ti. The fits to the minima using the creep law [7] are shown in red. Lower panel: Examples of Kerr microscopy images depicting the domain expansion in the two samples. Green arrows show the magnetization configuration within the domain wall while the red arrows depict the direction of the applied in-plane magnetic field.

Effect of limited spatial resolution of laser annealing

In our previous work [1], we have shown that the minimal magnetic feature that we can pattern by direct-write laser annealing is ~180 nm. To assess how the limit of the spatial resolution affects the lateral exchange coupling in devices variable lateral dimensions, we have investigated the influence of laser fluence and racetrack width on domain wall velocities. A strong inverse relationship between mean domain wall velocity and track width can be seen in Fig. S3: wider tracks consistently exhibit lower velocities, while narrower tracks support faster DW propagation. Laser fluence significantly influences the critical width required for non-zero domain wall velocity. Specifically, increasing the laser fluence shifts the velocity-width curve along the *x*-axis, leading to a reduction in domain wall velocities for a given track width.

The domain wall velocity in the creep regime in the absence of an external magnetic field can be expressed by

$$v = v_0 e^{-\eta(\mu_0 H_{\mathrm{EC}})^{-\frac{1}{4}}}, \qquad (2)$$

where v_0 is the characteristic speed, η is the scaling coefficient, which is proportional to the thermal energy. H_{LEC} is the effective exchange coupling field that drives the domain wall motion and can be expressed as:

$$\mu_0 H_{\mathrm{LEC}} = \frac{\lambda_{\mathrm{DW}}}{M_{\mathrm{tot}} w_{\mathrm{track}}}, \qquad (3)$$

where λ_{DW}, M_{tot}, w_{track} are the domain wall energy density, net magnetization, and width of the track. As the equation suggests, smaller track widths result in a larger effective exchange field. Since the domain wall velocity depends exponentially on H_{EC}, a higher velocity is expected in narrower tracks.

The fits to the measured velocities as a function of track width are shown in Fig. S3 for different laser fluences, assuming $\eta\ (M_{\mathrm{tot}}/\lambda_{\mathrm{DW}}) = \mathrm{const.}$ is the same for each laser fluence. The obtained fits, which have a reasonable correspondence with the experimental trends, suggest that M_{tot} and $\lambda_{\mathrm{DW}} \approx \sqrt{AK_{\mathrm{eff}}}$ can indeed be assumed to be constant. We thus conclude that the limited spatial resolution of the laser-patterning technique does not affect the device performance when scaled down from 8 μm to 2 μm.

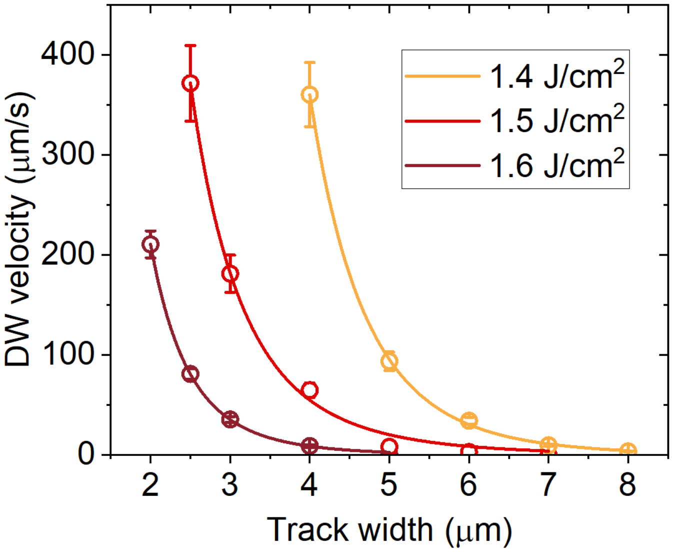

Figure S3*:* (a) Domain wall velocities as a function of the track width for various laser fluences. Fits (lines) to the experimental data (open symbols) are calculated using Equations (2) and (3).

Effect of temperature variations

To address the sensitivity of our devices to temperature variations, we have measured the domain wall velocity distributions at two different temperatures in devices with $w_{\mathrm{track}} = 3\ \mu\mathrm{m}$ and $w_{\mathrm{device}} = 20\ \mu\mathrm{m}$, as shown in Fig. S4. One can see a significant shift in the domain wall velocities when changing the temperature from 23°C to 33°C, which is given by the nature of the thermally assisted domain wall motion in the creep regime as well as by the sensitivity of the ferrimagnets to temperature variations.

From these results, it is therefore clear that one has to take the temperature dependent behavior into account for a particular application. One possibility is to work in the flow regime of the domain wall dynamics rather than in the creep regime since this would allow one to work in a less temperature sensitive regime. This can be achieved by further reducing the track width. To do this, we have previously shown that local oxidation can be achieved through plasma oxidation through a mask patterned by electron beam lithography [8]. In particular, we have shown that this can be reliably done to create tracks with lateral dimensions down to ~50 nm [9].

Nevertheless, the key requirement to obtain the lateral exchange coupling is that the two neighboring regions need to be above and below the compensation temperature. This requirement can be easily satisfied by using an as-grown film with the compensation temperature well above the room temperature, which makes the lateral exchange coupling more immune to temperature variations.

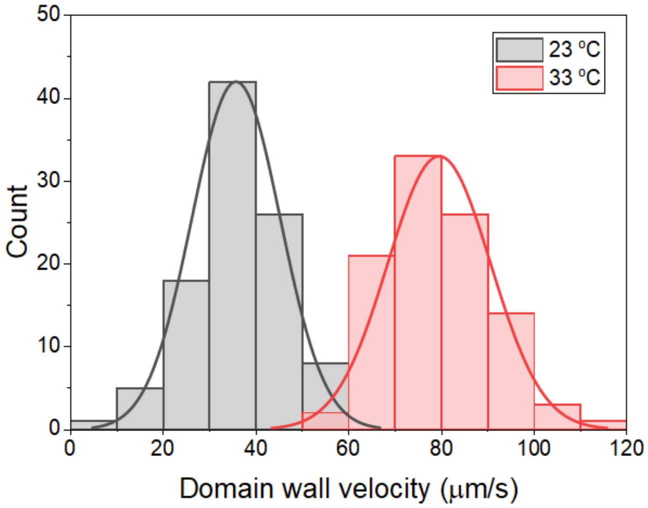

Figure S4*:* Distribution of spontaneous domain wall velocities at 23 °C (black) and at 33 °C (red). Here, the device is fabricated from $Gd_{30}Co_{70}$ films with $w_{track} = 3\ \mu m$ and $w_{device} = 20\ \mu m$. The tracks were annealed at a laser fluence of 1.97 J/cm^2. The statistics is based on 100 velocity measurements.

S4 Micromagnetic Simulations

Micromagnetic simulations were performed using the mumax^3 software package [10]. To model our materials, we have employed the following simulation parameters unless stated otherwise: Uniaxial perpendicular anisotropy constant K_u = 1 x 10^4 J/m^3, exchange stiffness *A* = 15 pJ/m, saturation magnetization M_s = 50 kA/m, interfacial Dzyaloshinskii-Moriya interaction (iDMI) energy density *D* = +0.55 mJ/m^2 and ferrimagnet thickness *t* = 1 nm. The simulated devices had a length of 2 μm and device widths w_{device} varying between 350 nm and 10 μm. The simulation geometry was discretized into 5 nm x 5 nm x 1 nm cells (*x*, *y*, and *z* directions, respectively). These coordinate axes are defined in Fig. S5. The simulations were initialized by setting the magnetization of a 200 nm-wide region at the center of the simulation, representing the TM-dominant track, along the -*z* direction, while the magnetization in the surrounding areas was oriented along the +*z* direction (Fig. S5a). Once the simulation began, LEC was sufficient to initiate domain wall motion starting from the apex of the simulated TM-dominant track (Fig. S5b-d). Unlike the experimental results shown in the main text, our simulations demonstrate an initial decrease in width of the domain in the simulated TM-dominant track once the simulation begins. This behavior stems from the fact that, in our simulations, the TM-dominant track and RE-dominant device have identical magnetic properties but opposing initial magnetic orientations. As such, our simulations do not account for DW pinning induced by the abrupt change in magnetic properties at the TM-RE boundary in our experimental devices [11–13]. Nevertheless, by *t* = 1 ns, the width of the TM-dominant region stabilizes. Accordingly, in our analysis of the simulation results, we only consider the system when *t* > 1 ns, where the TM-dominant region has reached a steady-state width.

Micromagnetic simulations were initially used to determine the relationship between *A* and the speed of DWs driven by LEC. Keeping all other magnetic parameters constant to the values stated above, *A* was varied between 1 pJ/m and 25 pJ/m. Periodic boundary conditions were applied along the ±*x*-directions in this simulation to mimic a semi-infinite RE-dominant region surrounding the TM-dominant track. We find that the DW leak and reset velocity ($v_{leak/reset}$)

exhibits a monotonic increase with A (Fig. S6), confirming that changing A provides a material-intrinsic means of tuning the speed of DW motion in response to LEC, as shown in Fig. 2e of the main text.

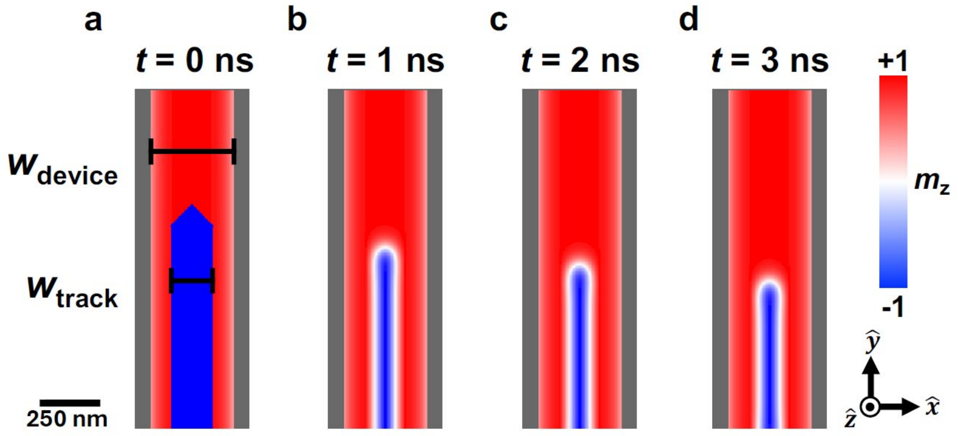

Figure S5: **(a)** Schematic of the normalized z-component of the magnetization in the initialized state and **(b)** 1 ns, (**c**) 2 ns, and (**d**) 3 ns after the micromagnetic simulation began for a system with w_{device} = 400 nm, illustrating the simulated DW motion in response to LEC.

To micromagnetically determine how LEC-induced DW motion is impacted by the presence of the chiral edge canting promoted by the iDMI, we then constrained the magnetization of the RE-dominant region surrounding the TM-dominant track to point along the +z-axis. In Figs. S7a,b (Figs. S7c,d), we show the simulated magnetization profile of systems with chiral edge canting enabled (disabled) and w_{device} values of 800 nm and 400 nm, respectively, 3 ns after the simulation began. In contrast to when chiral edge canting is enabled (Figs. S7a,b), a head-to-head arrangement between the magnetization of the DW and the edges of the device does not develop when chiral edge canting is disabled (Figs. S7c,d), as further demonstrated by the cross-sectional view shown in Fig. 3e of the main text (corresponding to the region indicated by the dotted red line in Fig. S7d). This approach, where we freeze the magnetic moments at the edges and we do not change iDMI, allows us to keep the overall energy scale similar since, otherwise, variations of iDMI energy density would affect the DW energy density [5-7].

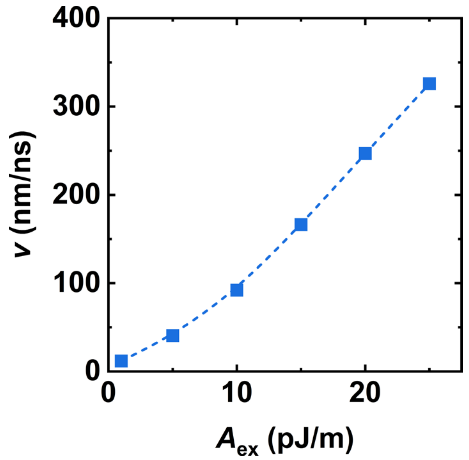

Figure S6: Simulated $v_{leak/reset}$ as a function of exchange stiffness A. The width of the simulated TM-dominant region was held constant at 200 nm. To simulate a semi-infinite film, periodic boundary conditions were applied along the x and y directions.

To understand how chiral edge canting of the magnetization affects the DW energy, and consequently the velocity of LEC-driven DWs, we examine the DW energy density ε_{DW} at the DW center in our simulations (location illustrated by the magenta dot in Fig. S7d), 3 ns after the simulations began. When chiral edge canting is suppressed, ε_{DW} remains constant with respect to w_{device} (red triangles in Fig. S7e). In contrast, when chiral edge canting and the associated partial head-to-head magnetic configuration are present (as in Fig. 3d of the main text), ε_{DW} decreases with increasing w_{device} (blue circles in Fig. S7e). This trend reflects the fact that increasing w_{device} also enlarges the separation between the DW and the canted edge states, thereby weakening their dipolar interaction. Comparing Fig. S7e with the DW velocity versus w_{device} data shown in Fig. 3c of the main text, the higher DW velocity at smaller w_{device} can be understood as follows: Because DWs with higher energy densities are less energetically favorable and LEC-induced reversal of the TM-dominant region removes the DW and its associated energy from the system, it is more energetically favorable for the TM-dominant region to switch in the case of narrower w_{device}, leading to faster LEC-driven DW motion.

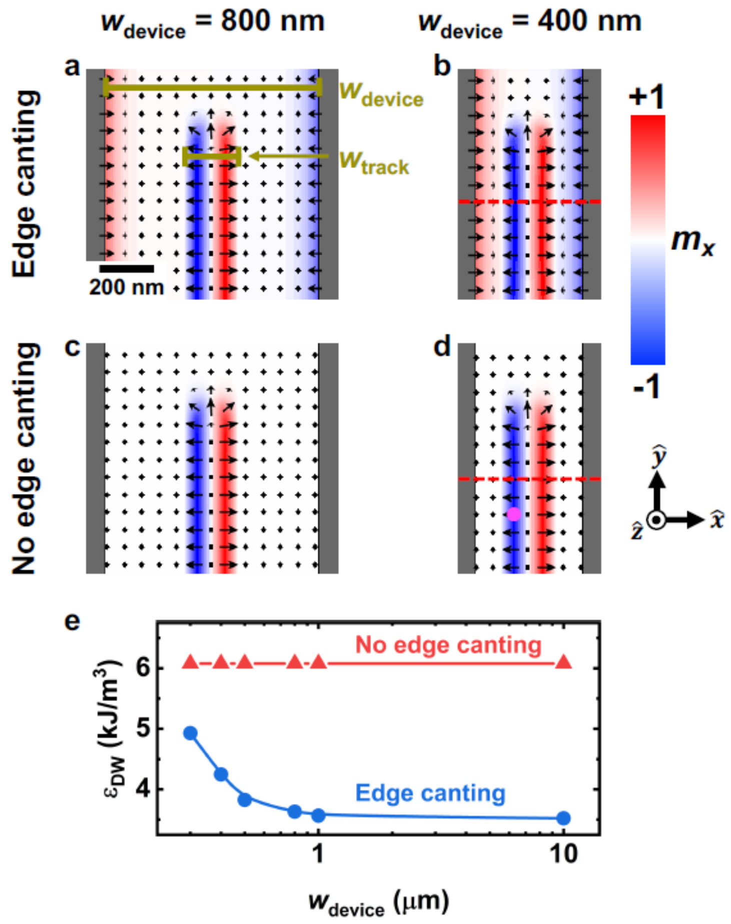

Figure S7: (a-d) Top-down depictions of the magnetic configuration of systems with (a,b) and without (c,d) chiral edge canting, and w_{device} values of 800 nm (a,c) and 400 nm (b,d), 3 ns after the simulation began. The simulated regions shown correspond to regions similar to the one enclosed by the blue box in Fig. 3a of the main text. The cross-sectional view shown in Fig. 3d (Fig. 3e) of the main text corresponds to the magnetization along the dashed red line in Fig. S7b (Fig. S7d). **(e)** The total micromagnetic energy density ε_{DW} in the center of the DW region [indicated by magenta dot in (d)], 3 ns after the simulation began for systems with (blue dots) and without (red triangles) edge canting for a range of w_{device} values.

S5 Supporting Video Captions

Supporting Video 1: Polar MOKE video of the domain evolution of the $Co_{70}Gd_{30}$ sample discussed in Fig. 2 of the main text. The video begins while a +200 mT out-of-plane magnetic field was applied to initialize the sample and tracks the evolution in the position of the DWs along the tracks after the magnetic field was set to zero. Details on the device design are provided in the main text.

Supporting Video 2: Polar MOKE video of the domain state present in the device shown in Fig. 3a of the main text. The video starts while a +200 mT out-of-plane magnetic field was applied to the sample and shows the evolution in the position of the DWs along the tracks once this magnetic field was removed.

Supporting Video 3: Polar MOKE video of the leaky integration and passive reset behavior of the neuronal structure shown in Fig. 4 of the main text as SOT current pulses are applied. The video begins after the initializing magnetic field has been removed and LEC-driven domain wall motion has occurred, resulting in the DW becoming pinned on the left side of the image where w_{track} becomes large enough to suppress further spontaneous DW motion. As electrical current pulses are applied, SOT-induced DW motion to the right corresponds to the integration of the input signal, whereas the LEC-driven DW motion to the left between SOT pulses mimics neuronal leaking. When the SOT current pulses are stopped 16.8 s into the video, LEC causes the DW to retreat to the left, towards the original position of the DW. Details on the SOT pulse characteristics are provided in the caption of Fig. 4 of the main text.

References

[1] Riddiford, L. J. *et al*. Two-dimensional gradients in magnetic properties created with direct-write laser annealing. Nature Communications 16, 10979 (2025).

[2] Caretta, L. *et al.* Fast current-driven domain walls and small skyrmions in a compensated ferrimagnet. *Nature Nanotechnology* 13, 1154 (2018).

[3] Honda, S. & Yoshiyama, M. Magneto-optical Kerr rotation and sublattice magnetic moments in RE-Co sputtered films. *Japanese Journal of Applied Physics* 27, 1687 (1988).

[4] Mangin, S. *et al.* Engineered materials for all-optical helicity-dependent magnetic switching. *Nature Materials* 13, 286 (2014).

[5] Pellegren, J. P. *et al.* Dispersive Stiffness of Dzyaloshinskii Domain Walls. *Physical Review Letters* 119, 027203 (2017).

[6] Lau, D. *et al.* Disentangling factors governing Dzyaloshinskii domain-wall creep in Co/Ni thin films using Pt_xIr_{1-x} seed layers. *Physical Review B* 98, 184410 (2018).

[7] Hrabec, A. *et al.* Measuring and tailoring the Dzyaloshinskii-Moriya interaction in perpendicularly magnetized thin films. *Physical Review B* 90, 020402 (2014).

[8] Liu, Z. et al. Strong lateral exchange coupling and current-induced switching in single-layer ferrimagnetic films with patterned compensation temperature. *Physical Review B* 107, L100412 (2023).

[9] Luo, Z. et al. Current-driven magnetic domain-wall logic. *Nature* 579, 214 (2020).

[10] Vansteenkiste, A. *et al.* The design and verification of MuMax3. *AIP Advances* 4, 107133 (2014).

[11]Van de Wiele, B., Hämäläinen, S. J., Baláž, P., Montoncello, F. & Van Dijken, S. Tunable short-wavelength spin wave excitation from pinned magnetic domain walls. *Scientific Reports* 6, 21330 (2016).

[12] Baláž, P., Hämäläinen, S. J. & Van Dijken, S. Static properties and current-induced dynamics of pinned 90 magnetic domain walls under applied fields: An analytic approach. *Physical Review B* 98, 064417 (2018).

[13] Franken, J. H., Hoeijmakers, M., Lavrijsen, R. & Swagten, H. J. Domain-wall pinning by local control of anisotropy in Pt/Co/Pt strips. *Journal of Physics: Condensed Matter* 24, 024216 (2011).
